# Increasing aggregate size reduces single-cell organic carbon incorporation by hydrogel-embedded wetland microbes

**DOI:** 10.1093/ismeco/ycae086

**Published:** 2024-06-20

**Authors:** Juliet T Johnston, Bao Nguyen Quoc, Britt Abrahamson, Pieter Candry, Christina Ramon, Kevin J Cash, Sam C Saccomano, Ty J Samo, Congwang Ye, Peter K Weber, Mari-Karoliina Henriikka Winkler, Xavier Mayali

**Affiliations:** Physical and Life Sciences, Lawrence Livermore National Laboratory, 7000 East Ave, Livermore CA 94550, United States; Civil and Environmental Engineering, University of Washington, 201 More Hall, Box 352700, Seattle, WA 98195-2700, United States; Civil and Environmental Engineering, University of Washington, 201 More Hall, Box 352700, Seattle, WA 98195-2700, United States; Civil and Environmental Engineering, University of Washington, 201 More Hall, Box 352700, Seattle, WA 98195-2700, United States; Physical and Life Sciences, Lawrence Livermore National Laboratory, 7000 East Ave, Livermore CA 94550, United States; Chemical and Biological Engineering, Colorado School of Mines, 1500 Illinois St, Golden, CO 80401, United States; Quantitative Biosciences and Engineering, Colorado School of Mines, 1500 Illinois St, Golden, CO 80401, United States; Chemical and Biological Engineering, Colorado School of Mines, 1500 Illinois St, Golden, CO 80401, United States; Physical and Life Sciences, Lawrence Livermore National Laboratory, 7000 East Ave, Livermore CA 94550, United States; Physical and Life Sciences, Lawrence Livermore National Laboratory, 7000 East Ave, Livermore CA 94550, United States; Physical and Life Sciences, Lawrence Livermore National Laboratory, 7000 East Ave, Livermore CA 94550, United States; Civil and Environmental Engineering, University of Washington, 201 More Hall, Box 352700, Seattle, WA 98195-2700, United States; Physical and Life Sciences, Lawrence Livermore National Laboratory, 7000 East Ave, Livermore CA 94550, United States

**Keywords:** hydrogel, nanoSIMS, stable isotopes, reactive transport modeling, nanosensors, sediment microbial ecology

## Abstract

Microbial degradation of organic carbon in sediments is impacted by the availability of oxygen and substrates for growth. To better understand how particle size and redox zonation impact microbial organic carbon incorporation, techniques that maintain spatial information are necessary to quantify elemental cycling at the microscale. In this study, we produced hydrogel microspheres of various diameters (100, 250, and 500 μm) and inoculated them with an aerobic heterotrophic bacterium isolated from a freshwater wetland (*Flavobacterium* sp.)*,* and in a second experiment with a microbial community from an urban lacustrine wetland. The hydrogel-embedded microbial populations were incubated with ^13^C-labeled substrates to quantify organic carbon incorporation into biomass via nanoSIMS. Additionally, luminescent nanosensors enabled spatially explicit measurements of oxygen concentrations inside the microspheres. The experimental data were then incorporated into a reactive-transport model to project long-term steady-state conditions. Smaller (100 μm) particles exhibited the highest microbial cell-specific growth per volume, but also showed higher absolute activity near the surface compared to the larger particles (250 and 500 μm). The experimental results and computational models demonstrate that organic carbon availability was not high enough to allow steep oxygen gradients and as a result, all particle sizes remained well-oxygenated. Our study provides a foundational framework for future studies investigating spatially dependent microbial activity in aggregates using isotopically labeled substrates to quantify growth.

## Graphical Abstract

**Graphical Abstract:**
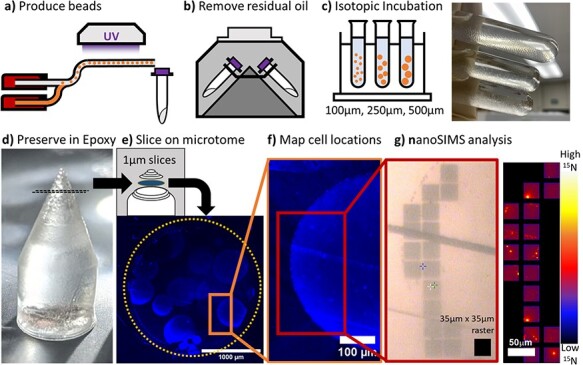

## Introduction

Sediment microbial communities process organic carbon (C) originating from photosynthetically fixed C from plants and algae. Wetlands are notably C rich, sequestering 20–30% of global soil C despite representing only 5–8% of terrestrial surfaces [1]. Tracking C in these ecosystems can be a challenge due to complex microbial biotransformation processes, redox gradients controlled by O_2_ availability, as well as the chemical and physical properties of sediments impacting diffusivity and permeability, including the size of aggregates [2]. To reduce this complexity in order to test hypotheses about the impact of aggregate size on wetlands C cycling processes, hydrogels provide a simple-to-fabricate, replicable, and size-controlled matrix for rapid and gentle entrapment of prokaryotes [3–7] to create micro-environments to simulate sediment particles [8]. Hydrogels, which are transparent and free of minerals, are also more compatible with microscopic and molecular techniques compared to natural sediment aggregates [9, 10]. This approach has enabled linking spatio-functional dynamics of C cycling microbes across particle size distributions which is key for a mechanistic understanding of C transformations [11–13] and determines the redox potential controlling microbial community composition and reaction energetics [8, 14–16]. Previous studies investigated larger diameters suitable for wastewater aggregates and coarse sand particles (500 to 5000 μm diameters [3, 7, 17, 18]), while our study aimed to focus on smaller particle size distributions in wetlands [19].

An increasingly utilized approach to measure microbial activity involves the addition of stable isotope labeled substrates to quantify anabolic activity [20]. Following incubations with isotope labeled substrates, samples can be analyzed at the bulk level, or alternatively, imaging mass spectrometry can maintain spatial information. Nanoscale secondary ion mass spectrometry (nanoSIMS) enables spatially resolved measurements of cellular incorporation of stable isotope labeled substrates, even in a complex system where the dispersion of a single originating substrate can be quantified after multiple biotransformation steps [21]. Tracking O_2_ concentrations with similar spatial micrometer resolution would provide useful information regarding microbial metabolism at the scale of individual aggregates. Although this cannot currently be performed in soils due to opacity, luminescent nanosensors - that can quantify O_2_ concentrations at the microscale - have recently been developed and are compatible with hydrogel microspheres [22, 23]. By embedding such sensors alongside microbial communities within these aggregates, O_2_ concentration gradients could be correlated with measurements of microbial C incorporation to better understand the effect of O_2_ concentration and C availability on microbial metabolism in aggregates [24, 25].

Here, we implemented a method that can correlate O_2_ availability and organic C incorporation at the microscale within aggregates, and we analyzed the impact of three hydrogel particle sizes on microbial activity using a bacterial isolate (*Flavobacterium sp.*) and a diverse wetland microbial community. We determined particle sizes of 100, 250, and 500 μm diameter as representative of sandy wetlands ranging between very fine sand, fine-medium fine sand, and coarse sand [19]. We hypothesized that smaller particles, which are less diffusion-limited due to higher surface-to-volume ratios, would exhibit higher per-volume microbial activity. We incubated the microbial cells with ^13^C labeled substrates to quantify C incorporation into cellular biomass using nanoSIMS while maintaining the spatial location of those cells during analysis. These data were correlated to O_2_ concentration as measured by embedded luminescent nanosensors. The results were further investigated by creating a reactive transport model to simulate long-term growth beyond our relatively short-term experiments.

## Materials and methods

### Experimental design overview

We carried out three experiments to confirm microbial growth and quantify bacterial incorporation on hydrogel microspheres. The first was a preliminary experiment to confirm microbial growth and activity within hydrogel microspheres, while the second and third were detailed follow-ups on microbial growth and activity with spatial resolution. The first experiment, without isotope labeled substrates, was performed with a single aerobic heterotrophic isolate (*Flavobacterium* sp.) from a freshwater lake sediment [26]. In this initial experiment, *Flavobacterium* sp. was incubated on M9 minimal media with 50 μM of glucose for 88-hours in sealed test tubes. At intervals (8, 32, 56, and 88 hours), small aliquots of hydrogel microspheres were removed for DAPI staining to quantify microbial growth via fluorescence intensity using z-stacks on a fluorescent confocal microscope.

After confirming microbial growth within a hydrogel microsphere, we carried out follow-up experiments with isotope labeled substrates, first using the same isolate (*Flavobacterium* sp.) and second with a diverse mixed wetland microbial community. These experiments were designed to spatially quantify the incorporation of isotopically labeled substrate into biomass and dissolved oxygen concentration within the hydrogel microspheres. Prior to hydrogel encapsulation, *Flavobacterium* sp. was pre-cultured in R2A liquid media [27] with 50 μM ammonium chloride. The wetland community was encapsulated directly after extraction from wetland sediment. Microbial cells were encapsulated in spherical hydrogel microspheres (see below) of three different sizes (100, 250, and 500 μm diameter) to examine the influence of aggregate size on population and single cell metabolism. Approximately 1 mL hydrogel microsphere solution was used in biological triplicates with 9 mL of media and incubated in sealed test tubes at room temperature on a shaker table at 30 rpm. The encapsulated *Flavobacterium* was first grown for 48-hours in R2A media with ^15^N labeled ammonium, washed, incubated for 24-hours in M9 minimal media with no C substrate, before a 24-hour isotopic incubation in M9 minimal media with 500 μM ^13^C-glucose as the sole C source. Glucose was selected for the *Flavobacterium* as a simple sugar commonly utilized by most organisms to generate a fundamental baseline for our investigation. The mixed community was incubated in lacustrine media (described in the Supplemental Materials) with 500 μM of ^13^C-labeled and ^15^N-labeled algal protein (Sigma-Aldrich, Cat No: 642878 and 586773) and an additional 50 μM of ^15^N-ammonium (Cambridge Isotopes Item No. NLM-107-PK) for a total of 96-hours. Algal protein was selected as a commonly available but more complex C substrate requiring multiple pathways within the diverse wetland community to consume. At the end of the experiments, 5 mL of gaseous headspace was extracted for measurement of ^13^CO_2_ gas respired, while microspheres were fixed with 4% paraformaldehyde for downstream processing.

### Wetland cell extraction

A complex microbial community was extracted from a wetland soil sample taken from an urban lacustrine wetland (University of Washington Arboretum, Seattle, WA, USA—47° 38′ 31.12” N, 122° 17′ 47.01” W). Samples were taken using a 2” × 4” soil corer (AMS, American Falls, ID, USA) driven down 60 cm. Cores were transported to the lab on wet ice and stored at 4°C until processing. Cells were extracted in an anaerobic tent (Coy Lab Products, Grass Lake, MI, USA) to minimize potential oxygen toxicity. Soil was first suspended (10% w/v) in lacustrine medium, followed by sieving (2 mm) to remove coarse particles. The sieved soil suspension was then paddle blended (Stomacher® 400 Circulator, Seward, UK) for 3 x 1 min at 250 rpm with 1-min rests between runs. Subsequently samples were centrifuged at 200 g for 2 min to remove coarse organics and filtered through a 20 μm filter (Whatman™ Grade 41, Cytiva, Marlborough, MA, USA) to remove eukaryotes and fungal material. Cell extracts were stored in anaerobic bottles (N_2_:CO_2_ 80:20) at 4°C until the start of the experiment.

### Gas measurements

Respiration of C into ^12^CO_2_ and ^13^CO_2_ was measured by collecting 5 ml of liquid media for isotopic quantification using a Picarro G2201 -i Cavity Ring Down Spectrometer (Picarro Inc, Santa Clara, CA, USA). Collection vials were initially sealed with one atmosphere of gaseous headspace before autoclaving. After 5 mL of liquid media was added, samples were acidified using 0.33 mL of 1 N HCl to dissociate bicarbonate into carbon dioxide during a 24-hour equilibration. Each vial then had 5 mL of gaseous headspace removed using a gastight syringe injected into the Small Sample Introduction Module 2. The factory protocol was then run with a zero-air dilution and analyzed for ^13^CO_2_/^12^CO_2_. Ambient air was used between every fifth injection as a reference point for calibration and to ensure lack of instrument drift. CH_4_ was below the limit of detection (<1 ppm).

### Oxygen measurements

The concentrations of dissolved O_2_ within hydrogel microspheres were measured using PtTFPP/DiA oxygen nanosensors [23]. This was achieved by creating a batch of hydrogel microspheres where the mixture of polyethylene glycol diacrylate (PEGDA) and cells was diluted 50% using a 0.2 μM filtrate of PtTFPP/DiA sensors (filtration is required to avoid adding aggregated sensors). Controls included incubations with no nanosensors, kill controls with saturated O_2_ levels, and no-O_2_ controls with 10 units · mL^−1^ of glucose oxidase with 10 mM glucose. The batches of hydrogel microspheres with the O_2_ nanosensors were incubated in near-identical conditions to the previously described experimental batch with isotope labeled substrates, and micrographs were taken on a Nikon W1 SoRa Spinning Disk Confocal Microscope (Nikon, Tokyo, Japan) using the 405 nm excitation with 435–485 nm emission as the reference fluorescent signal and 405 nm excitation with 640–660 nm emission as the O_2_ concentration fluorescent signal. Single cross sections were used to normalize the intensity of signals into ratios. Images were processed using ImageJ by dividing the O_2_ fluorescent signal by the reference signal. The saturated O_2_ killed control and the anaerobic glucose oxidase control were then used to calculate a Stern-Volmer standard curve to interpolate the O_2_ concentration across different fluorescent ratio measurements as previously described [23].

### Hydrogel microsphere formulation

Hydrogel microspheres were made by mixing 4.5 mL of cell culture with 0.5 mL PEGDA Mn700 and 0.1 mg of Lithium phenyl-2,4,6-trimethybenzoyphospinate as a photoinitiator [28] from Sigma Aldrich (St Louis, Missouri, USA) by light vortexing. Microspheres were formed by running this mixture through a syringe pump passing from a blunt needle tip, into clear tubing with steady flowing carrier fluid of Xiameter^TH^ PMX-200 Silicone Fluid 10cst and 2% v/v of DOWSIL™ RSN-0749 RSN from DOW Chemicals (Midland, Michigan, USA). The carrier fluid and hydrogel microspheres passed under a 365 nm UV lamp for 10–15 seconds of exposure for the photoinitiator to solidify the PEDGA hydrogel into a spherical microsphere, which were collected in a 30 μm pluriStrainer filter from pluriSelect (Leipzig, Germany). The pluriStrainers were then slowly centrifuged at 4°C for 2-min at 1000 rfc to remove the residual silicone oil. A schematic of this design and photographs of this process are shown in the supplemental materials.

To achieve different diameter hydrogel microspheres, different needle gauges, tubing diameters, and flow rates of both the carrier fluid and cell culture+ PEDGA mixture determined the diameter of the microspheres. For example, 500 μm diameter was achieved with a 26 g blunt needle, PEGDA flow rate of 3 mL/hour, oil flow rate of 21 mL/hour into 0.6 mm inner diameter tubing. Two hundred and fifty μm diameter was achieved with a 30 g blunt needle, PEGDA flow rate of 1.5 mL/hour, oil flow rate of 6 mL/hour 0.42 mm inner diameter tubing. One hundred μm diameter was achieved with a 32 g blunt needle, PEGDA flow rate of 1 mL/hour, oil flow rate of 12 mL/hour into 0.25 mm inner diameter tubing.

### Quantifying microbial growth in 3D structures

The *Flavobacterium* sp. cells were embedded into hydrogel microspheres and then incubated over an 88-hour period using M9 minimal media supplemented with 50 μM glucose as the sole C source. Minimal media was used to minimize fluorescence interference from potential precipitants. Conventional methods to measure growth such as OD600 and fluorescence intensity on a 96-well plate reader were inconsistent due to the opacity of the hydrogel. Instead, we performed confocal microscopy with a Nikon W1 SoRa Spinning Disk Confocal Microscope (Nikon, Tokyo, Japan) at 405 nm excitation with 435–485 nm emission to measure DAPI fluorescence intensity through the beads over time. Each microsphere had up to 30 z-stacked images to reconstruct the entire 3D structure and we compared average fluorescence intensity across time.

### NanoSIMS preparation and analysis

Formalin-fixed microspheres were dehydrated in 100% ethanol in a 70°C incubator for 8 hours. Once dehydrated, the microspheres were then embedded into LR White Epoxy Resin (Ted Pella, Redding, California, USA) using the manufacturer’s protocols except for the successive infiltration steps [29]. Infiltration or LR White was carried out over 5-dilution mixtures starting with 20% mixture of LR White in 80% ETOH, then 50%, 80%, and 2x 100% infiltrations. The embedded microspheres were then sliced into 1 μm thin slices using a diamond blade on a Leica Reichert Ultracut S microtome (Wetzlar, Germany). The slices were then stained with DAPI and mapped for cell locations using a Nikon W1 SoRa Spinning Disk Confocal Microscope (Nikon, Tokyo, Japan). After mapping, the slices were lightly washed with milliQ water to remove residual DAPI, air dried, placed on glass slides and gold coated to increase surface conductivity.

To analyze the assimilation of organic C into microbial biomass, ^13^C and ^15^N isotope incorporation was measured in individual cells using the CAMECA NanoSIMS 50 at Lawrence Livermore National Laboratory. Four masses (^12^C_2_^−^, ^12^C^13^C^−^, ^12^C^14^N^−^, and ^12^C^15^N^−^) were measured using electron multipliers at a mass resolving power of ~7000 [1.5× correction [21]]. Each location targeted by the nanoSIMS had a raster size of 35 μm × 35 μm and was scanned for 25 cycles which were then composited into a single mosaic image using L’Image software (developed by L Nittler, Carnegie Institution of Washington, Washington, DC, USA). Individual cells were extracted in L’image as hand-drawn regions of interest (ROIs) based on the ^12^C^14^N^−^ or ^12^C^15^N^−^ images. Individual cell distance from the surface of a hydrogel microsphere was calculated using a combination of the distance measurements mapped by fluorescence microscopy and by coordinate geometry using the location of three cells detected on the outer surface of a microsphere to calculate the center of the sphere and radial distance of nearby cells. Cells from incubations with isotope labeled substrates were determined to be active or inactive by comparing to no-addition control cells and were required to be 2 standard deviations above the average isotope composition of no-addition control cells.

Assimilation of C into biomass was calculated using C_net_ [30, 31] (Equation 1), which uses the fraction of ^13^C found in cells incubated with a ^13^C labeled substrate (C_f_), unlabeled control cells for the background isotope ratio (C_i_) and the fraction of ^13^C found in the media (C_s_). Due to a previously identified 5-fold dilution of ^13^C from the resin embedding [32], C_net_ values were then multiplied by a factor of 5 (not shown in the equation).


(1) \begin{equation*} {\boldsymbol{C}}_{\boldsymbol{net}}=\frac{{\boldsymbol{C}}_{\boldsymbol{f}}-{\boldsymbol{C}}_{\boldsymbol{i}}}{{\boldsymbol{C}}_{\boldsymbol{s}}-{\boldsymbol{C}}_{\boldsymbol{i}}} \end{equation*}

### Modeling

One-dimensional reactive-transport models of the hydrogel microspheres were constructed to evaluate C and O_2_ consumption resulting from the growth and decay of either a *Flavobacterium* isolate or a wetland community comprised of three representative species embedded within the hydrogels. Individual microspheres were modeled as a one-dimensional, fixed thickness, spherical biofilm with a distinct diameter (100, 250, and 500 μm) incubated in a well-mixed environment with various concentration of organic C and a fixed O_2_ concentration in the bulk liquid. Solute concentrations were set at constant values at the biofilm surface assuming a fully aerated bulk liquid (8 mg-O_2_/L) and a constant C concentration (1, 50, 100, 200, 500, 1000 µmM-C). The fixed thickness assumption was appropriate given the fixed diameter of the manufactured hydrogel microspheres.

The model for aerobic C consumption within the hydrogel microspheres was based on the material balances for C and oxygen. The time-dependent mole balances for all soluble components within the spherical hydrogels included net reaction rates resulting from microbial C and oxygen consumption. The isolate model considered the aerobic growth of *Flavobacterium sp.* on glucose as the sole C source. The growth rate of *Flavobacterium sp.* was measured in this study, while other kinetic parameters were obtained from the literature [33–38]. The wetland community model considered the aerobic growth of three representative heterotrophic species (a growth strategist, a yield strategist, and an average organism) on a single C substrate instead of a mixture of C substrates. Neither the formation of products from the incomplete substrate consumption nor byproduct mediated cross-feeding between community members were considered in the wetland community model. The single C substrate assumption was appropriate to model the competition for the ^13^C labeled algal protein measured in this study. Since the composition of the wetland community was unknown, the selection of species representing a heterotrophic growth specialist, heterotrophic yield specialist, and an average heterotroph was appropriate based on previous studies evaluating substrate competition within microbial communities [39–43]. The growth rate of the representative wetland species was based on parameters obtained from the literature [33–38].

The model for biomass production and decay were based on the mole balance for individual hydrogel microsphere components. It was assumed that the hydrogel microspheres were comprised of active biomass, inert biomass, and a cross-linked hydrogel polymeric matrix, which was modeled as inert biomass. The modeled hydrogels were assumed to have a constant density of 50 mg/L with an initial uniform distribution of biomass [37]. A time-dependent mole balance for all hydrogel components included biomass growth, biomass decay, and the advective transport of all biomass components resulting from biomass growth.

A zero-flux boundary condition for all soluble components was set at the center of the hydrogel. Mass-transfer resistance at the hydrogel surface was neglected for all solutes, causing solute concentrations at the hydrogel surface to be equivalent to the bulk liquid. Based on the assumption of a constant bulk environment, the bulk liquid was not modeled. Initial concentrations throughout the hydrogel were assumed to be equivalent. Steady-state behavior within the hydrogels was evaluated after simulating biomass growth and decay over a period of 300 days. The model was implemented and evaluated in COMSOL Multiphysics (v4.4 Comsol Inc, Burlington, MA). Detailed methods and assumptions along with all model equations, process matrix, and a complete list of parameters are provided in Supplemental Material (Supplementary Tables S1–S7).

## Results and discussion

### Method development

**Figure 1:**
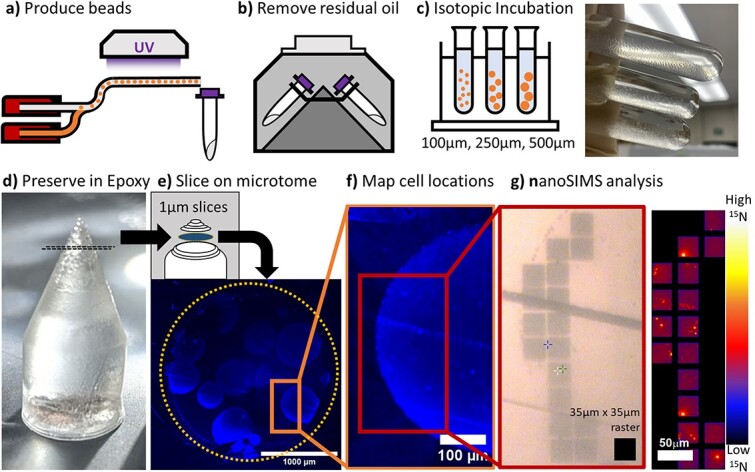
Workflow to generate hydrogel-embedded microbial communities for stable isotope tracing coupled with nanoSIMS: (a) hydrogel microspheres (“beads”) are produced by flowing PEGDA, photoinitiator LAP, and cell solution into silicone oil and hardened under UV 365 nm lamp. (b) The residual oil is removed by centrifugation with a pluriStrainer (30 μm mesh) at 1000 rcf for 2 min. (c) The isotope addition experiment is performed on different diameter PEDGA microspheres (100, 250 and 500 μm). (d) After the experiment, fixed cells in the hydrogel microspheres are preserved in LR white epoxy so that they can (e) be sliced into 1 μm thin fragments on a microtome. The slices are stained with DAPI and imaged with fluorescence microscopy to identify the location of cells in relation to the microsphere surface (f) for later isotope analysis in the nanoSIMS (g).

We developed a protocol (Fig. 1) to quantify location-specific microbial C uptake in hydrogel microspheres via incubation with stable isotope labeled C under controlled laboratory settings followed by nanoSIMS analysis. We first applied our approach in experiments with a single bacterial isolate (*Flavobacterium* sp.) growing on a single C source (glucose), and subsequently applied the method to a complex microbial community extracted from a wetland growing on protein as the C source. We produced hydrogel microspheres of three diameters (Fig. 1a and b) with embedded microbial cells, which allowed them to be incubated in various liquid media and rinsed without losing the cells in the process. At the conclusion of the incubation, the hydrogel-embedded cells were fixed with formaldehyde to stop cellular activity. Afterward, multiple hydrogel microspheres from the same incubation were preserved in resin to maintain the spatial arrangement of the cells within each microsphere (Fig. 1d). The resin provides a dense casing so that the beads can be thinly sliced (Fig. 1e) for downstream microscopy and subsequent isotope imaging analysis (Fig. 1f and g). Microscopy provides coordinate locations of the cells to link cell-specific growth with distance from the microsphere-liquid interface.

### Microbial growth in hydrogel microspheres

**Figure 2:**
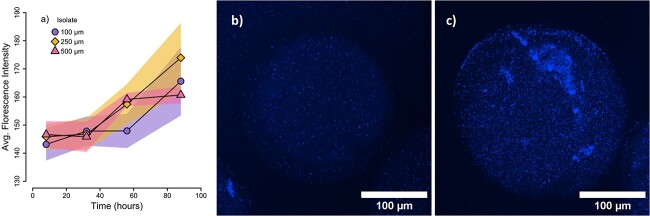
To confirm bacterial growth in hydrogel microspheres, a preliminary experiment using isolate *Flavobacterium* sp. was performed without isotope labeling. Bacterial growth was detected using DAPI staining at each timepoint. The DAPI fluorescent signal was calculated across z-stacked average fluorescence intensity with a confocal microscope for replicate hydrogel microspheres. (a) Quantification of growth for the isolate *Flavobacterium* sp. within hydrogel microspheres of respective sizes 100 μm (circles), 250 μm (diamonds), and 500 μm (triangles) grown on M9 minimal media with glucose. Each timepoint represents the average while the shaded region represents the standard deviation of five microspheres. Representative maximum intensity projection images are shown from two 250 μm diameter microspheres at the 8-hour (b) and 56-hour (c) timepoints, respectively showing increased fluorescence signal at the later timepoint.

After encapsulation in hydrogel microspheres, we confirmed growth of *Flavobacterium* sp. with glucose as the sole C source (Fig. 2). We first measured the fluorescence intensity inside the microspheres after staining with a DNA-binding dye 8-hours after inoculation and found no statistically significant differences across the three diameter sizes (Students t-test, *P* > 0.5). This lag growth period continued through the first 32-hours (56 hours for the 100 μm microspheres), but fluorescence subsequently increased through the 88-hour last time point. The longer lag time compared to suspended cultures is likely a result of the microsphere synthesis process that puts physiological stress on cells during transfer between the silicon carrier fluid, UV exposure during photoinitiation, and acclimation into new media. Additionally, some of the initial fluorescence signal likely originated from dead cells and residual DNA from the suspended culture. Similar long lags have been observed in comparably sized 100–300 μm microspheres inoculated with *Escherichia coli* as well as much larger 2500–5000 μm diameter aggregates with ammonia oxidizing archaea [3, 7, 44]. For subsequent experiments, we ensured a sufficient acclimation period to promote cellular activity prior to incubation with isotope labeled substrates. We also confirmed respiration of isotopically labeled C into ^13^CO_2_ via isotopic gas measurements (Supplementary Fig. S1).

### Microsphere size impacts microbial growth

We first examined the growth of microbial cells encapsulated in hydrogel microspheres of different sizes without examining their distance from the microsphere surface to gather a population-level understanding of metabolism within the aggregates, similar to what a bulk analysis might generate. We hypothesized that either or both O_2_ and C availability might limit growth, and thus we expected that cells in the smaller microspheres might grow faster than cells in larger ones, since smaller aggregates should be less diffusion limited. To quantify growth, we calculated C_net_, which represents the fraction of cellular C derived from the substrate (glucose in the first experiment and protein in the second experiment) [45]. Low activity and inactive or dead cells were found in both experiments (see Supplementary Table S7 for total and active cell numbers), and the populations showed heavily positively skewed distributions (Supplementary Fig. S2a,c). Skewness occurs when the probability distribution function is uneven, and our high positive skewness (range 0.9–1.43) demonstrates that most of the cells had low isotope incorporation, with a small percentage of cells having high activity.

A further examination of the population distribution of activity across microsphere sizes suggests that smaller microsphere size was indeed associated with greater activity. To statistically test for differences across size, we compared the cumulative distribution functions of the different sized microspheres (Supplementary Fig. S2b,d), which calculate the probability that a cell in the population has an activity level equal to or greater than a given value. Using Kolmogorov–Smirnov tests, we found that the populations in the 100 μm microspheres were statistically different than the populations in the 250 and 500 μm microspheres in both experiments (isolate Kolmogorov–Smirnov 100 μm–250 μm D = 0.469, 100 μm–500 μm D = 0.315, 250 μm–500 μm D = 0.332, all *P* < <0.05, and wetland Kolmogorov–Smirnov 100 μm–250 μm D = 0.138 *P* < <0.05, 100 μm–500 μm D = 0.134 *P* < <0.05 and 250 μm–500 μm D = 0.079 & *P* = 0.280). This means that a randomly sampled cell from the smaller microspheres had a statistically significantly higher chance of having higher growth than a cell sampled from the other two microsphere sizes. This result suggests that growth in smaller microspheres was faster than in larger ones, presumably due to lower diffusion limitation in the small microspheres.

Although our experiments examined C incorporation, we also incubated the microspheres with ^15^N labeled ammonia and protein for the wetland experiment in order to have an independent measure of growth. We found a linear relationship between ^15^N and ^13^C enrichment for all microsphere sizes (Supplementary Fig. S3), and these N incorporation data showed similar positive skewness as the C_net_ data (Supplementary Fig. S4). The ^13^C enrichment values were adjusted by a factor of 5 due to the known 5-fold dilution that occurs from the LR white resin embedding process due to unlabeled C from the resin infiltrating into the cells [21, 32]. While positively skewed distributions are common in nanoSIP studies [45–47], the overall C_net_ was lower than expected when compared to similar length incubations of planktonic cells [48–50]. In addition to the possibility that the cells might have incorporated some C from the hydrogel polymer, future studies will require positive controls (100% ^13^C labeled cells) to quantifiably resolve the dilution occurring from the resin embedding process, or if using CARD-FISH to target specific microbes of interest [51–53].

### Aggregate size influences depth-activity relationship

**Figure 3:**
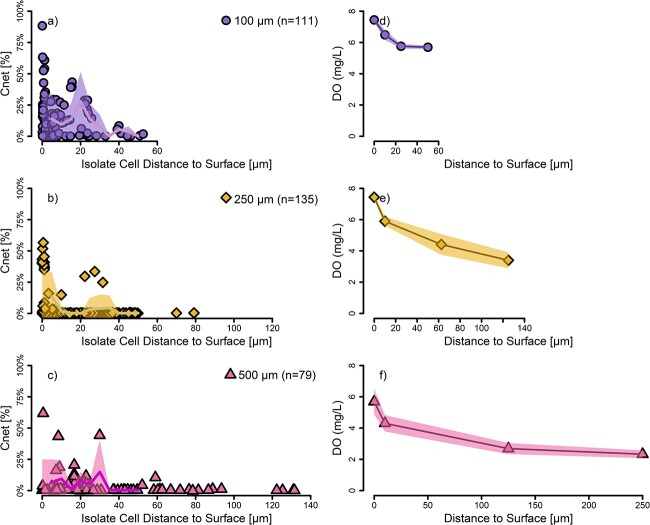
Growth as measured by C incorporation for *Flavobacterium* sp. and dissolved oxygen content in hydrogel microspheres of three difference sizes (100, 250, and 500 μm) as a function of depth. Growth, C_net_ = %, is the bacterial biomass originating from stable-isotope labeled glucose (a, b, c). Points are individual cells. A moving average at 5 μm increments is represented by the solid lines and shaded areas correspond to the standard deviations of data. The oxygen concentrations showed a corresponding decrease in oxygen (d, e, f) and was calculated from fluorescence intensity of microsphere-embedded nanosensors from a separate incubation under similar conditions.

We next examined the cell-specific growth data in the context of spatial organization within and across microsphere sizes for both experiments to constrain the impact of aggregate size on microbial activity. Consistent with diffusion-limited growth, the highest activity by *Flavobacterium* occurred at or near the surface of the microspheres for all three sizes, with a general decline going deeper into the center (Fig. 3). Within the first 5 μm from the surface, there was no statistically significant differences between bacterial cells from the three microsphere sizes (Kruskal–Wallis *P* = 0.52). However, considering cells within the first 50 μm from the periphery of the microsphere, there was a statistically significant effect of diameter (Kruskal–Wallis *P* < < 0.05). A *post hoc* Tukey HSD test showed that the 100 μm microspheres were comprised of more active cells than the other two sizes. Beyond 50 μm depth, the bacterial cells in the 250 and 500 μm diameter microspheres exhibited low activity (Fig. 3). These results indicate that small aggregates may allow microbially mediated transformations of organic matter to progress more quickly, which might be due to higher surface area to volume ratio compared to larger particles.

**Figure 4:**
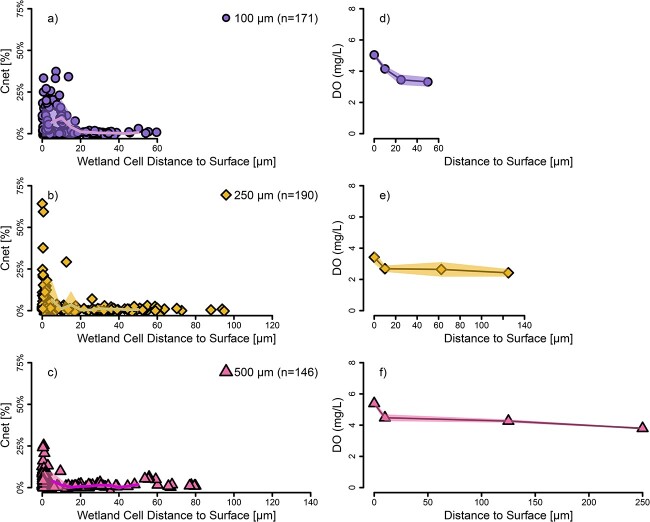
Wetland microbial community growth as measured by C incorporation and dissolved oxygen content in hydrogel microspheres of three difference sizes (100, 250, and 500 μm) as a function of depth. Growth, C_net_ = %, is the bacterial biomass originating from stable-isotope labeled protein (a, b, c). Points are individual cells. A moving average at 5 μm increments is represented by the solid lines and shaded areas correspond to the standard deviations of data. The oxygen concentrations showed a corresponding decrease in oxygen (d, e, f) and was calculated from fluorescence intensity of microsphere-embedded nanosensors from a separate incubation under similar conditions.

Similar to the *Flavobacterium* experiment, the three microsphere sizes for the wetland community showed equivalent single cell activity within the first 5 μm near the surface (Fig. 4a–c; Kruskal–Wallis *P* = 0.0859), and with increasing depth to 50 μm, microsphere diameter had a statistically significant impact on single cell activity (Kruskal–Wallis *P* = 0.0196). In this experiment, the 100 μm microspheres were significantly more active within the first 50 μm than the 500 μm microspheres (Tukey HDS *P* = 0.0182), while the 250 μm microspheres were not significantly different than the 100 μm (*P* = 0.129) or the 500 μm (*P* = 0.652) microspheres. Our initial hypothesis was that there would be similar organic C availability as a function of depth regardless of particle size, which would then result in equivalent bacterial growth as a function of depth. We partially rejected this hypothesis in two independent experiments, finding cells embedded in the smaller particles exhibiting significantly higher C uptake than those in larger particles, even if they were located at the same distance from the surface. A previous study that examined the microbial community structure of natural particles noted two distinct groups based on size (≥125 μm and < 125 μm) [54], and another study identified that different proteins for organic matter degradation were expressed deeper in the particles compared to closer to the surface [55]. These studies suggest that community structure differences could explain our results for the wetland community experiment. However, the *Flavobacterium* experiment only included one organism, so community differences could not explain those differences in activity. Thus, both community composition and aggregate size may be important drivers of organic matter processing in wetland environments. Further investigations will be needed to understand what physico-chemical differences occur that lead to these differences in activity in order to accurately model C utilization in particles of different sizes [56].

In both experiments but especially in the *Flavobacterium* experiment, we identified spikes of high activity between 20 and 40 μm into the microspheres (Fig. 3). We also observed dense volumes of microbial cells within the hydrogel microspheres suggesting larger microporous cavities caused by incomplete crosslinking (Supplementary Fig. S5). Macropores are larger porous spaces that form throughout the skeletal structure of hydrogels and can be created either intentionally for higher porosity or result due to incompletely crosslinked materials during polymerization [57, 58]. If macropores were connected to the outside of the microsphere, this would be a physical mechanism to increase diffusion of growth substrates from the outside, rather than having to diffuse through the hydrogel matrix. While it is unclear why these activity spikes were not as pronounced in wetland community incubations (Fig. 4), this could simply be a result of heterogeneity across samples or of diffusion limitations of the larger proteins that might first need to be metabolized into smaller compounds before reaching the macropores [59].

### Aggregate oxygen gradients- higher at surface to lower in the core without reaching anoxia

To correlate single cell C incorporation inside the aggregates to environmental conditions, we carried out similar incubation experiments of hydrogel-embedded *Flavobacterium* and wetland microbes with embedded O_2_ nanosensors. These experiments revealed a spatially dependent O_2_ gradient with higher concentrations near the surface and lower concentrations toward the microsphere core while never becoming anoxic (Fig. 3d–f, Fig. 4d–f, Supplementary Fig. S6) or even microaerobic [60]. We note that O_2_ concentrations at the microsphere surface were not equivalent in the incubations with different microsphere sizes. Variations in O_2_ concentration at the interface between the microsphere surface and the liquid were likely a result of respiration from planktonic cells not embedded in the hydrogel in combination with cells attached to the microsphere surface. Planktonic cells were inevitable since they could escape from the hydrogels due to incomplete crosslinking, the gel microstructure, or cells breaking open the matrix as they grew. While undesirable, cells escaping into the planktonic phase is a prevalent problem in hydrogel microsphere encapsulation studies [9]. Thus, we focused our analyses on the changes in O_2_ concentrations throughout the microsphere rather than absolute concentrations.

In all experiments and microsphere sizes, O_2_ concentrations decreased more sharply near the microsphere surface and for the larger microspheres, stabilized at deeper depths. We focused our analysis on the first 50 μm from the microsphere surface to examine if there were any differences in the rate of O_2_ concentration decrease across aggregate size (Supplementary Fig. S7a and b). We found that the O_2_ gradient over the first 50 μm in the *Flavobacterium* experiment did not show much difference across microsphere sizes (Supplementary Fig. S7a). However, the gradient in the 100 μm microspheres for the wetland experiment was steeper and statistically significantly different than the other sizes (Tukey HSD 100 μm–250 μm *P* = 0.002 & 100 μm–500 μm *P* = 0.008). This revealed that the 100 μm microspheres had a sharper O_2_ gradient within the first 50 μm compared to the 250 and 500 μm microspheres. The steeper change in O_2_ gradient in the smaller microspheres with the wetland community (but not with the isolate) suggests that a multi-species community is likely to be more active at breaking down organic C and further processing it hence consuming more oxygen, and this activity near the surface is greater for smaller aggregates compared to larger ones. Similarly to the higher C incorporation rates found in the smaller microspheres, these O_2_ gradient data again suggest that smaller aggregates exhibited greater activity near their surface, though it was not detected in the *Flavobacterium* experiment. Unique O_2_ gradients in small particles have previously been predicted by models [4, 5], and while other studies have utilized similar oxygen nanosensors [61–63], we are not aware of other studies having directly documented gradient differences in such small particles.

### Reactive transport modeling confirms microbial activity controlled by C availability

**Figure 5:**
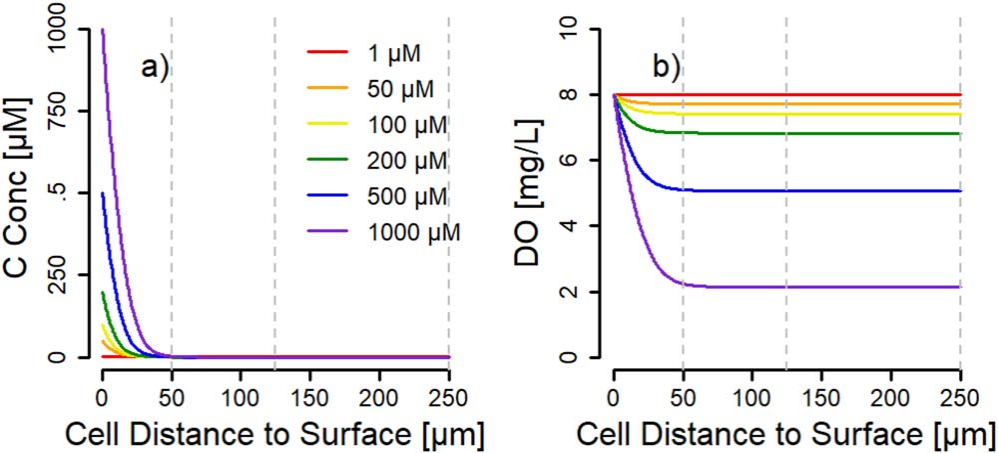
One-dimensional modeling of microbial consumption of organic carbon from protein (a) and dissolved oxygen (b) as function of depth in microspheres inoculated with a 3-member microbial community. Dotted lines represent the location of the center of 100, 250, and 500 μm diameter microspheres (simulations gave identical results in the first 50 μm from the surface for all three sizes). The modeled concentration of organic carbon supplied to the microsphere surface ranged from 1 to 1000 μM (see legend). The model suggests that the organic carbon supply was limiting, and the microspheres never reached oxygen limiting conditions at steady state. The model was built on a 300-day simulation of steady-state supply of carbon and oxygen to determine the gradients across microsphere diameters.

We computationally simulated the *Flavobacterium* sp. and wetland experiments using a one-dimensional, fixed thickness, spherical hydrogel reactive transport model for the three microsphere diameters tested in this study. These simulations allowed us to evaluate the impact of substrate limitation by altering the external C concentration, as well as examining long-term steady state behavior of microspheres incubated for 300 days in an oxygenated, well-mixed liquid medium with a constant substrate concentration. The isolate and wetland community simulations revealed that the hydrogel microspheres experienced organic C limitation leading to fully aerobic aggregates when continuously supplied with a constant concentration of C for 300 days. Across different organic C concentrations in the liquid medium (from 1 μM up to 1000 μM; 500 μM was used in the experiments), organic C was limiting within the first 50 μm into a microsphere at steady-state after 300 days of simulated incubations, for both the *Flavobacterium* experiment with glucose (Supplementary Fig. S8) and the wetland experiment with protein (Fig. 5). Cellular activity and O_2_ concentrations of the simulated experiments correspond to and reinforce the experimental results, with decreasing active biomass concentrations from the surface to inside the microspheres (Supplementary Fig. S9), corresponding to decreasing O_2_ concentrations without reaching anoxia. The simulations were unable to replicate the experimental results demonstrating an increase in C utilization with decreasing microsphere diameters when comparing the C assimilation of cells located at similar distances from the aggregate periphery. This difference may be caused by the experimental microspheres having imperfections, such as cracks and pores, which would allow more rapid penetration of the organic C, and potentially those imperfections were more significant for the smaller microspheres than larger ones. The differences in C utilization could also be attributed to the cross-feeding of metabolic byproducts between microbial community members in the microsphere experiments since the models only evaluated the consumption of a single C substrate.

Our experimental and simulation results suggest that embedding microbial cells collected from wetland sediments in hydrogel microspheres behave similarly to biofilms and can be used to investigate microbially mediated processes within and on aggregates. Regardless of microsphere size, all experiments and simulations indicate most C assimilation occurred within the first 50 μm from the microsphere periphery, reflecting the 2–10 μm thick biofilms sediment associated microbes typically form around the surface of soil particles and the rapid C decomposition rates observed in sediments with pore diameters between 15 and 90 μm [64–67]. Utilizing hydrogel microspheres as an experimental model system that can closely simulate conditions occurring in natural sediments could increase our understanding of soil particle associated microbial processes. For example, our experiments were carried out in oxic conditions, and the simulations assumed consistently saturated O_2_ levels outside of the microspheres. Developing experimental strategies to manipulate O_2_ concentrations in space (e.g. within an individual microsphere and across microsphere layers within a bioreactor) and time (e.g. fluctuation, drought-rewetting cycles) could approximate the conditions observed in the field where O_2_ concentrations and redox potential are a function of depth below the water table [8, 25, 68]. Similarly, embedding particulate organic C inside the microspheres could simulate the presence of organic C in natural sediment aggregates. Dynamically changing O_2_ concentrations below the water table will likely result in more complex carbon–oxygen interactions as anaerobic communities break down organic C substrates with different mechanisms and resulting efficiencies.

Individual and multi-scale aggregate models have been applied to accurately predict the influence of environmental conditions on the distribution of microbial populations and metabolites within wetland sediment aggregates and biofilm aggregates in wastewater treatment facilities [36, 38, 69–72]. Aggregates within these environments experience dynamic redox conditions that influence both the distribution of microbial processes within individual aggregates and bulk activity of the system. An individual sediment aggregate model that considered nitrifying, denitrifying, and aerobic C-degrading microorganisms was developed to quantify the impact of aggregate size and sediment depth on microbial community distribution and greenhouse gas production rates [73, 74]. Results from those studies suggest aggregate size and pore diameter exert significant influence over the distribution of aerobic and anaerobic metabolisms with both smaller sediment diameters and larger pore diameters favoring aerobic metabolisms. Although anaerobic conditions were not observed in our study, our experimental and modeling results confirm the relationship between particle size and aerobic activity. Smaller synthetic aggregates were found to have higher microbial cell-specific growth per volume and absolute activity near the aggregate periphery compared to larger particles. Additionally, the results from individual wetland aggregate models have been used to develop equations that describe the influence of aggregate size and environmental concentrations on greenhouse gas production rates [75]. By parameterizing the processes occurring within individual aggregates into an analytical equation, those previous studies were able to incorporate the influence of individual aggregates (μm scale) on greenhouse gas emissions at a regional ecosystem scale (km scale process). A similar approach could also be employed to incorporate the results from experiments conducted with hydrogel synthetic sediment aggregate into regional multi-scale wetland models. Individual aggregate models (μm scale) based on the results of hydrogel synthetic sediment aggregate experiments could be directly coupled to the diffusive flux of nutrients within the subsurface at defined spatial intervals (mm – m scale) and regional sediment types (km scale) though the computational complexity of these models would need to be taken into consideration [36, 38, 69–72]. While future mathematical models integrating environmental concentrations, individual aggregates, and ecosystem properties may be more applicable to modeling real-world wetland conditions, our current simulations strongly reinforce the experimental results and strengthen the validity of our stable isotope substrate methodological approach to unravel the complexities of spatially arranged microbial communities.

### Implications for microbial ecology research in wetlands

Here we demonstrated that our hydrogel microsphere encapsulation approach enabled us to link microbial C incorporation and oxygen concentration to spatial location within aggregates. Our simplified system has the potential to be scaled up to simulate a more complex sediment ecosystem, which could be constructed with sediment columns in flow-through bioreactors, with an anoxic bottom and an aerated top. Additionally, future experiments could include microspheres seeded with particulate organic materials such as cellulose or lignin to simulate microbial growth on decaying plant material. Alternatively, mineral particles can be co-immobilized with microbial cells inside the microspheres to investigate mineral-organic matter interactions within aggregates. Small variations such as these offer endless opportunities for investigations to uncover how microscale elemental cycling and redox gradients impact microbial biogeochemistry in wetland sediments.

## Supplementary Material

aggregate_size_supplemental_ycae086

## Data Availability

All data are provided in the source data file, with the raw nanoSIMS images available upon request.
