# Supplementary material for: Increasing aggregate size reduces single-cell organic carbon incorporation by hydrogel-embedded wetland microbes
Source: ISME Commun. 2024 Jun 20;4(1):ycae086. doi: 10.1093/ismeco/ycae086 (PMC11227278; doi:10.1093/ismeco/ycae086)
Supplement: aggregate_size_supplemental_ycae086 [file aggregate_size_supplemental_ycae086.pdf]

# Supplementary Material

## Lacustrine Media

1. 10 mL salt stock A
2. 10 mL salt stock B
3. 1 mL SL-10
4. 1 mL Se-W
5. 0.1 mL 7-vitamin solution

<u>Salts solution stock A (100x); in 100 mL</u>

1. $CaCl_2.2H_2O$ – 0.359 g
2. $MgCl_2.6H_2O$ – 0.332 g
3. $NH_4Cl$ – 0.076 g

<u>Salts solution stock A (100x); in 100 mL</u>

1. $NaHCO_3$ – 0.11 g
2. $KHCO_3$ - 291 µL 0.1 M stock
3. $K_2HPO_4$ - 35.4 µL 1M stock

<u>SL-10 (1000x); in 1000 mL</u>

1. HCl (25%; 7.7 M) 10.00 ml
2. FeCl2 x 4 H2O 1.50 g
3. ZnCl2 70.00 mg
4. MnCl2 x 4 H2O 100.00 mg
5. H3BO3 6.00 mg
6. CoCl2 x 6 H2O 190.00 mg
7. CuCl2 x 2 H2O 2.00 mg
8. NiCl2 x 6 H2O 24.00 mg
9. Na2MoO4 x 2 H2O 36.00 mg

<u>Se-W (1000x); in 1000 mL</u>

1. NaOH: 0.05g,
2. $Na_2SeO_3$ x 5 $H_2O$: 3 mg
3. $Na_2WO_4$ x 2 $H_2O$: 4 mg

<u>7-vit (10,000x); in 100 mL</u>

1. Vitamin B12 100.00 mg
2. p-Aminobenzoic acid 80.00 mg
3. D(+)-Biotin 20.00 mg
4. Nicotinic acid 200.00 mg
5. Calcium pantothenate 100.00 mg
6. Pyridoxine hydrochloride 300.00 mg
7. Thiamine-HCl x 2 H2O 200.00 mg

# Schematic of Hydrogel Microsphere Development

A needle was secured into tubing with plastic bonder and then sterilized.

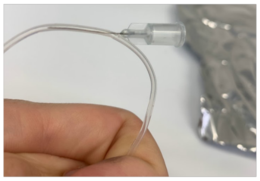

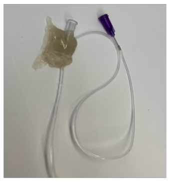

The two luer lock clips were connected to syringes containing 1) silicone oil as the carrier fluid and 2) cell culture + PEGDA + LAP. The syringes were then hooked up to syringe pumps.

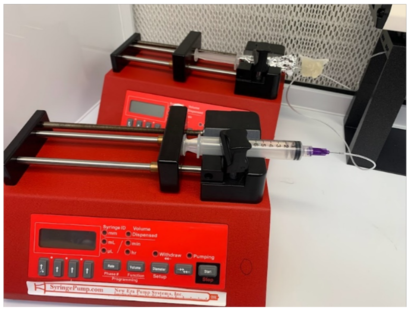

The tubing was run under a UV lamp to harder the PEGDA and cell cultures with the photoinitiator LAP.

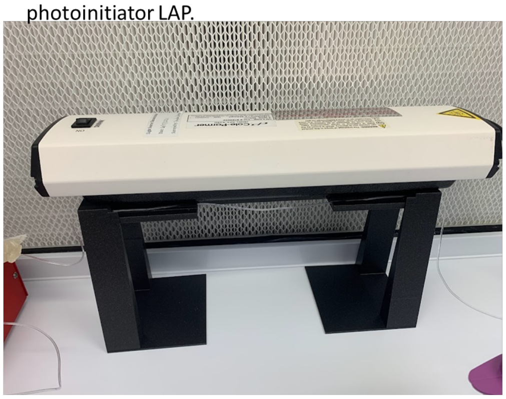

Hardened beads were separated from the silicone oil by pluricup strainers lightly spun on a centrifuge before being washed with fresh media.

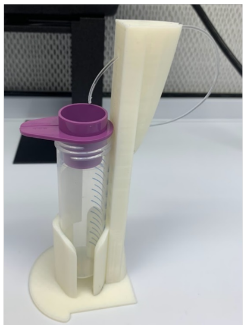

Methods: Numerical Modeling

The model for bacterial growth and decay within a hydrogel microsphere was based on the diffusion- reaction material balances for the relevant chemical species in the hydrogel exposed to a constant carbon ($C_{B,S}$) and oxygen ($C_{B,O2}$) concentration. A one-dimensional hydrogel with a fixed thickness ($L_F$) and a spherical geometry was assumed in all cases. The hydrogel was assumed to contain both biomass and a cross-linked polymeric matrix, represented by inert biomass. The isolate model only considers the growth of *Flavobacterium sp.* on glucose with biomass initially uniformly distributed throughout the hydrogel matrix ($X_i$). The wetland community model considers the growth of three species on a carbon substrate with equivalent amounts of biomass for all three species uniformly distributed throughout the hydrogel matrix ($X_i$). The only difference between the one species and three species models were the kinetic parameters (Table S1-4) and initial biomass concentrations.

The conversion rate of a component is related to the stoichiometric coefficient ($A_{ij}$) and the process rates to ($\rho_j$) through the equation $u_i=\sum_j A_{ij\rho}\, \rho_j$. The index $i$ refers to model components, while processes were marked by index $j$. Two types of components were present in the system, soluble ($S$) and particulate ($X$) substances[1].

## 1. Solute balances in the biofilm

Two model solutes (index i) were included in the model, with concentrations $C_{F,i}$ (mol/m3 biofilm): dissolved oxygen ($O_2$) and glucose (S). Time-dependent mole balances for all solutes in the hydrogel included rates of reaction and transport by diffusion, eq. (1):

$$\frac{\partial C_{F,i}}{\partial t} = D_{F,i}\nabla^2 C_{F,i} + r_i$$

with $\nabla^2 \equiv \frac{\partial^2}{\partial x^2} + \left(\frac{2}{x}\right)\frac{\partial}{\partial x}$ for the spherical hydrogel geometry.

The effective diffusion coefficients in the biofilm, $D_{F,i}$, were chosen based on values reported in Stewart 2003[2]. The net rates for each soluble component, $r_i$, were based on the stoichiometry and kinetics of carbon and oxygen consumption of each organism. Rates, kinetic expressions, stoichiometry, parameters are detailed in Tables S1-6.

The boundary condition at the hydrogel center (x=0) was set as zero-flux for all solutes, eq. (2):

$$D_{F,i}\left(\frac{\partial C_{F,i}}{\partial x}\right)_{x=0} = 0$$

The external mass transfer resistance (diffusion boundary layer) was neglected for all solutes. Therefore, concentrations at the hydrogel surface ($x=L_F$) were assumed to be equivalent with the bulk liquid concentrations, which were set to a constant value, eq. (3):

$$\left(C_{F,i}\right)_{x=L_F} = C_{B,i}$$

with $C_{B,i}$ in $mol/m^3_{liquid}$.

## 2. Biomass balances in the hydrogel

Biomass within the hydrogels were modeled as a fixed thickness biofilm containing active biomass ($X_i$), inert biomass ($X_{inert}$), and a cross-linked hydrogel polymeric matrix, which was represented by inert biomass ($X_{inert}$) in the model[3]. A constant hydrogel matrix density of was assumed for the hydrogel granule [3]. Biomass was assumed to be uniformly distributed throughout the hydrogel matrix and comprise 1% of the total hydrogel matrix. Biomass decay was described by the death-regeneration concept and was assumed to be a fixed fraction (10%) of the growth rate[4-6]).

A time-dependent mole balances for all biomass component in the hydrogel including reaction rates and f, eq. (4):

$$\frac{\partial X_i}{\partial t} = -u_F \frac{\partial X_i}{\partial x} + r_i$$

The net rates for each biomass component, $r_i$, were based on the stoichiometry and kinetics of carbon and oxygen consumption of each organism. Rates, kinetic expressions, stoichiometry, parameters are detailed in Tables S1-6.

The advective velocity of the hydrogel matrix within the hydrogel was computed based on the total hydrogel matrix mass balance, eq. (5).

$$\frac{\partial u_F}{\partial x} = \frac{\sum r_i}{\rho_{hydrogel}}$$

The boundary condition at the hydrogel center (x=0) was set as zero-flux for all biomass components, eq. (6):

$$\left(\frac{\partial X_i}{\partial x}\right)_{x=0} = 0$$

The boundary condition at the edge of the biofilm ($x=L_F$) was set as an outflow boundary condition, where biomass growing past the edge of the hydrogel was assumed to exit the hydrogel.

## 3. Model solution

The model was implemented in COMSOL Multiphysics (v4.4, Comsol Inc., Burlington, MA). Model equations were solved with variable time step on a biofilm domain discretized with a maximum mesh size of 1 μm. The simulation times were in the order of seconds per case. All reported steady state results were in all conditions obtained after 300 days[7].

**Table S1: Stoichiometric matrix of conversion reactions for** *Flavobacterium sp.* model

| $A_{ij}$ i component → <br> j process ↓ | $O_2$ [mM] | S [mM] | $X_i$ [mM] | $X_{inert}$ [mM] |
|---|---|---|---|---|
| **Biomass Growth** | | | | |
| 1. growth of *X* | - $Y_{O2/X}$ | - $Y_{S/X}$ | 1 | |
| **Biomass Decay** | | | | |
| 2. decay of *X* | | | -1 | 1 |

**Table S2: Stoichiometric matrix of conversion reactions for the three species wetland community** model

| $A_{ij}$ i component → <br> j process ↓ | $O_2$ [mM] | S [mM] | $X_1$ [mM] | $X_2$ [mM] | $X_3$ [mM] | $X_{inert}$ [mM] |
|---|---|---|---|---|---|---|
| **Biomass Growth** | | | | | | |
| 1. growth of species 1 | - $Y_{O2/X}$ | - $Y_{S/X}$ | 1 | | | |
| 2. growth of species 2 | - $Y_{O2/X}$ | - $Y_{S/X}$ | | 1 | | |
| 3. growth of species 3 | - $Y_{O2/X}$ | - $Y_{S/X}$ | | | 1 | |
| **Biomass Decay** | | | | | | |
| 4. decay of species 1 | | | -1 | | | 1 |
| 5. decay of species 2 | | | | -1 | | 1 |
| 6. decay of species 3 | | | | | -1 | 1 |

**Table S3: Kinetic rate expressions for** *Flavobacterium sp.* model

| **Reactions** <br> j process ↓ | Reaction Rate Equations |
|---|---|
| 1. Biomass growth | $r_1 = \mu_{max} * \frac{c_{F,S}}{K_S + c_{F,S}} * \frac{c_{F,O2}}{K_{O2} + c_{F,O2}} * c_{F,X}$ |
| 2. Biomass Decay | $r_2 = \mu_{max} * b_X * c_{F,X}$ |

**Table S4: Kinetic rate expressions for the** three species wetland community model

| **Reactions** <br> j process ↓ | Reaction Rate Equations |
|---|---|
| 1. Biomass growth (Species 1) | $r_1 = \mu_{max,1} * \frac{c_{F,S}}{K_{S,1} + c_{F,S}} * \frac{c_{F,O2}}{K_{O2} + c_{F,O2}} * X_1$ |
| 2. Biomass growth (Species 2) | $r_2 = \mu_{max,2} * \frac{c_{F,S}}{K_{S,2} + c_{F,S}} * \frac{c_{F,O2}}{K_{O2} + c_{F,O2}} * X_2$ |

| | |
|---|---|
| 3. Biomass growth (Species 3) | $r_3 = \mu_{max,3} * \frac{c_{F,S}}{K_{S,3} + c_{F,S}} * \frac{c_{F,O2}}{K_{O2} + c_{F,O2}} * X_3$ |
| 4. Biomass Decay (Species 1) | $r_4 = \mu_{max} * b_X * X_1$ |
| 5. Biomass Decay (Species 2) | $r_5 = \mu_{max} * b_X * X_2$ |
| 6. Biomass Decay (Species 3) | $r_6 = \mu_{max} * b_X * X_3$ |
| 7. Overall Biomass Decay | $r_7 = r_4 + r_5 + r_6$ |

**Table S5: Modeling Parameters for values** *Flavobacterium sp.* Model

| Parameter | Value | Unit | Description | Reference |
|---|---|---|---|---|
| **Stoichiometric parameters** | | | | |
| $Y_{O2/X}$ | 0.58 | $mol_{O2}/g_X$ | Stoichiometric value for yield coefficient on oxygen | Henze et al. 2006 |
| $Y_{S/X}$ | 1.58 | $mol_S/g_X$ | Stoichiometric value for yield coefficient on carbon | Henze et al. 2006 |
| **Kinetic parameters** | | | | |
| $\mu_{max}$ | 0.0719 | $\mathbf{h^{-1}}$ | Maximum growth rate in R2A media | Measured by this study |
| $K_{O2}$ | 0.5 | µM | Saturation coefficient for oxygen | Picioreanu et al. 1997 |
| $K_S$ | 0.054 | mM | Half saturation coefficient for carbon | Henze et al. 2006 |
| $b_X$ | 0.1 | | Decay ratio | Henze et al. 2006 |
| **Diffusion coefficients** | | | | |
| $D_{O2}$ | 2e-9 | $m^2/s$ | Diffusion coefficient for oxygen | Stewart 2003 |
| $D_S$ | 0.5e-9 | $m^2/s$ | Diffusion coefficient for carbon source | Stewart 2003 |
| **Boundary Conditions** | | | | |
| $C_{B,S}$ | 1, 50, 100, 200, 500, 700, 800, 1000 | µM | Carbon substrate concentration in the bulk | Chosen by this study |
| $C_{B,O2}$ | 0.25 | mM | Oxygen concentration in the bulk | Chosen by this study |
| **Biofilm Parameters** | | | | |
| $L_F$ | 50, 125, 250 | µm | Biofilm radius | Chosen by this study |
| $\rho_{hydrogel}$ | 50 | g/L | Biofilm Density | Typical value; Wanner et al. 2006 |
| $M_x$ | 113 | g/mol | Molar Mass of hydrogel matrix (assumed to be equivalent to biomass) | Typical value |
| $C_{F,X}$ | 353.98 | mM | Biomass / hydrogel matrix concentration | Calculated |
| **Initial Values** | | | | |
| $C_{o,X}$ | 0.01* $C_{F,X}$ | mM | Initial biomass concentration | Chosen by this study |
| $C_{o,Xi}$ | 0.99* $C_{F,X}$ | mM | Initial hydrogel matrix concentration | Chosen by this study |
| $C_{o,S}$ | 0 | mM | Initial glucose concentration | Chosen by this study |
| $C_{o,O2}$ | 0 | mM | Initial oxygen concentration | Chosen by this study |

**Table S6: Modeling parameters for** three species wetland model

| Paramete | Value | Unit | Description | Reference |
|---|---|---|---|---|
| **Stoichiometric parameters** | | | | |
| $Y_{O2/X}$ | 0.58 | $mol_{O2}/g_X$ | Stoichiometric value for yield coefficient on oxygen | Henze et al. 2006 |
| $Y_{S/X}$ | 1.58 | $mol_S/g_X$ | Stoichiometric value for yield coefficient on carbon | Henze et al. 2006 |
| **Kinetic parameters** | | | | |
| $\mu_{max,1}$ | 0.111 | $h^{-1}$ | Maximum growth rate of wetland community on algal proteins | Measured by this study |
| $\mu_{max,2}$ | 0.0719 | $h^{-1}$ | Maximum growth rate of *Flavobacterium sp.* on R2A media | Measured by this study |
| $\mu_{max,3}$ | 0.03 | $h^{-1}$ | Maximum growth rate | Chosen by this study |
| $K_{O2}$ | 0.5 | µM | Saturation coefficient for oxygen | Picioreanu et al. 1997 |
| $K_{S,1}$ | 0.07 | mM | Half saturation coefficient for carbon | Chosen by this study; Henze et al. 2006 |
| $K_{S,2}$ | 0.054 | mM | Half saturation coefficient for carbon | Chosen by this study; Henze et al. 2006 |
| $K_{S,3}$ | 0.02 | mM | Half saturation coefficient for carbon | Chosen by this study; Henze et al. 2006 |
| $b_X$ | 0.1 | | Decay ratio | Henze et al. 2006 |
| **Diffusion coefficients** | | | | |
| $D_{O2}$ | 2e-9 | $m^2/s$ | Diffusion coefficient for oxygen | Stewart 2003 |
| $D_S$ | 0.5e-9 | $m^2/s$ | Diffusion coefficient for carbon source | Stewart 2003 |
| **Boundary Conditions** | | | | |
| $C_{B,S}$ | 1, 50, 100, 200, 500, 700, 800, 1000 | µM | Carbon substrate concentration in the bulk | Chosen by this study |
| $C_{B,O2}$ | 0.25 | MM | Oxygen concentration in the bulk | Chosen by this study |
| **Biofilm Parameters** | | | | |
| $L_F$ | 50, 125, 250 | µm | Biofilm radius | Chosen by this study |
| $\rho_{hydrogel}$ | 50 | g/L | Biofilm Density | Typical value; Wanner et al. 2006 |
| $M_x$ | 113 | g/mol | Molar Mass of hydrogel matrix (assumed to be equivalent to biomass) | Typical value |
| $X_T$ | 353.98 | mM | Total Biomass / hydrogel matrix concentration | Calculated |

**Table S7: Initial values for** three species wetland model

| Paramete | Value | Unit | Description | Reference |
|---|---|---|---|---|
| **Initial Values** | | | | |
| $C_{o,Xi}$ | $0.01* X_T$ | mM | Initial biomass concentration | Chosen by this study |
| $C_{o,Xi}$ | $0.97* X_T$ | mM | Initial hydrogel matrix concentration | Chosen by this study |
| $C_{o,S}$ | 0 | mM | Initial glucose concentration | Chosen by this study |
| $C_{o,O2}$ | 0 | mM | Initial oxygen concentration | Chosen by this study |

| | Size | Total Cells Analyzed | Active Cells | Percent Active |
|---|---|---|---|---|
| **Isolate** | **100 µm** | 111 | 108 | 97.3% |
| | **250 µm** | 135 | 115 | 85.2% |
| | **500 µm** | 79 | 75 | 94.9% |
| | **Mixed Control** | 28 | - | - |
| **Wetland** | **100 µm** | 170 | 147 | 86.5% |
| | **250 µm** | 175 | 154 | 88.0% |
| | **500 µm** | 146 | 131 | 89.7% |
| | **Mixed Control** | 21 | - | - |

**Table S8** The number of cells analyzed per hydrogel microsphere size category, and how many cells as well as the percentage of cells which were statistically more active than the no-addition control cells.

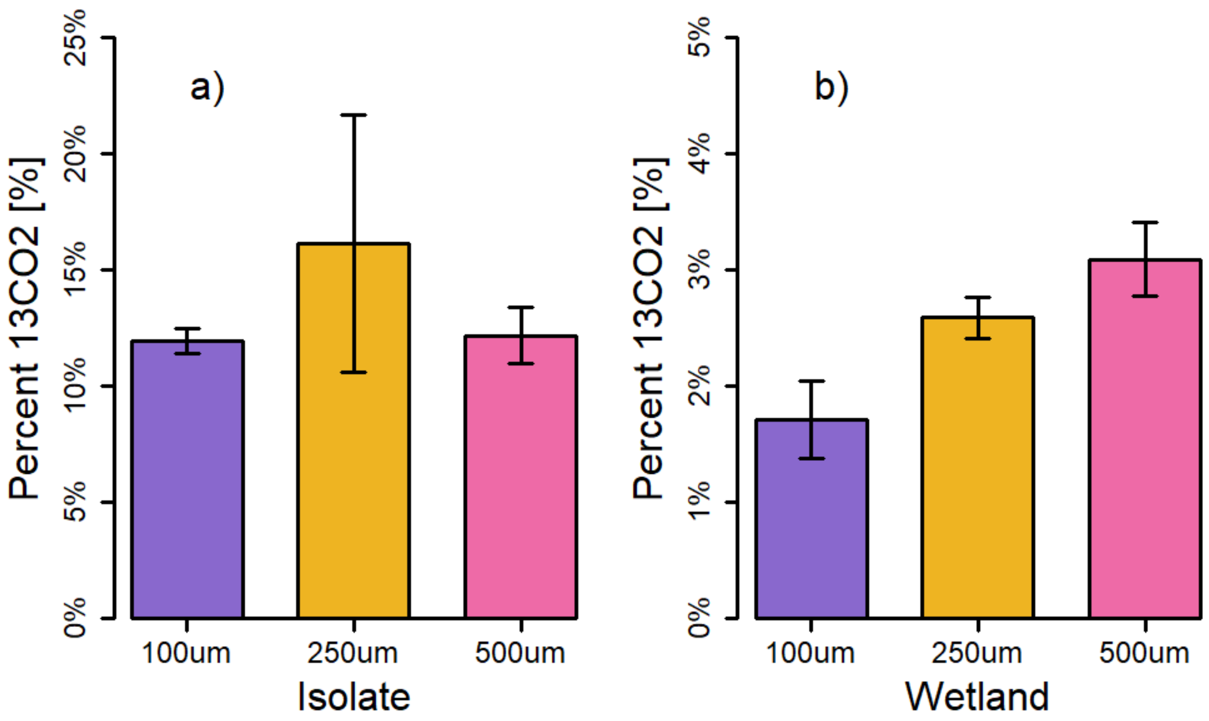


**Figure S1:** Picarro isotopic gas analysis showing $^{13}CO_2$ was generated from the *Flavobacterium* experiment growing on $^{13}C$-labeled glucose and the wetland microbial community experiment growing on $^{13}C$ labeled protein.

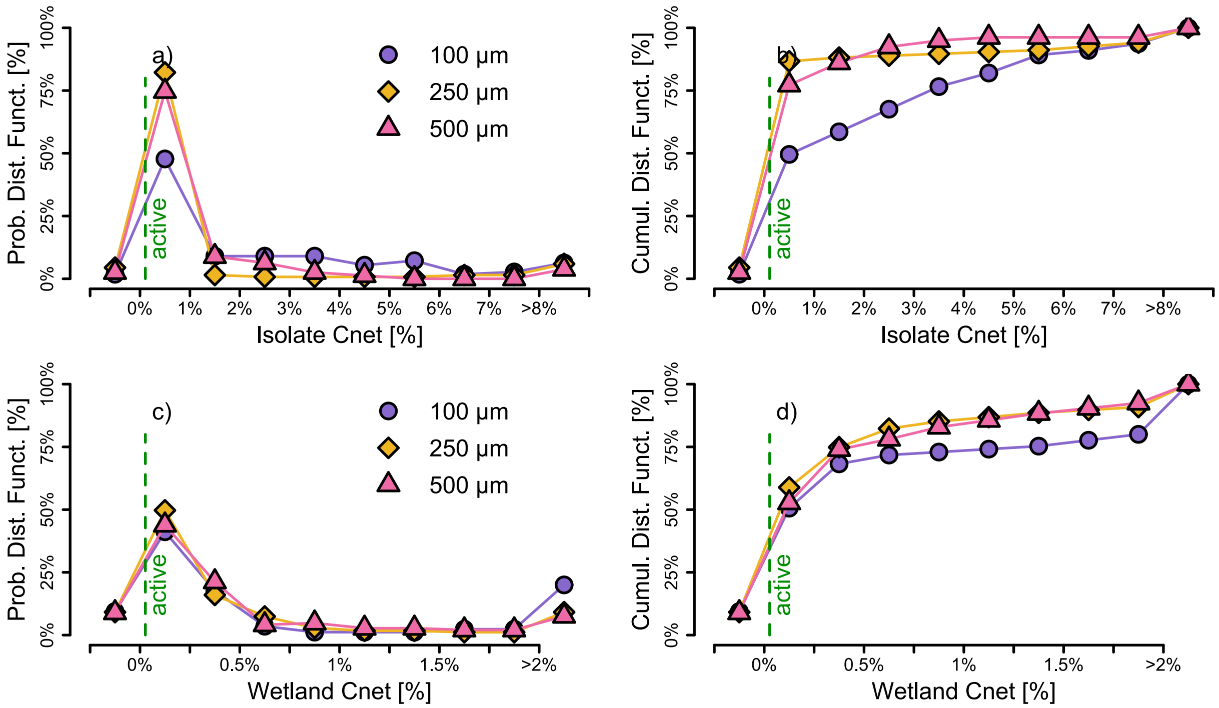


**Figure S2:** Isolate and wetland cells are more likely to be highly active in 100 µm microspheres using probability statistics. Inactive and low activity cells create a positively skewed distribution across all microsphere sizes, yet 100 µm spheres had a higher proportion of active cells compared to larger sizes. The a & c) probability distribution functions are the statistical likelihood of a cell being within a specific $C_{net}$ range, and the b & d) cumulative distribution functions are the likelihood of a cell having a $C_{net}$ at that percent or lower. The *Flavobacterium* (a &b) and wetland community (c & d) analyses are shown with 100µm micropheres (purple circles), 250µm (yellow diamonds) and 500µm microspheres (pink triangles). Overall, the mean of cells in 100 µm microspheres were statistically more enriched for $^{13}C$ than the larger 250µm and 500µm microspheres that were not significantly different from each other. The size data for the isolate experiment resulted in distinct cumulative distribution functions (Figure S2b) using Kolmogorov-Smirnov tests (100µm-250µm D=0.469, 100µm-500µm D=0.315, 250µm-500µm D=0.332, all p<<0.05). The wetland community's cumulative distribution functions (Figure S2d) showed more highly active cells in 100 µm microspheres resulting in a distinct distribution (100 µm-250µm D=0.138 & p<<0.05, 100 µm-500 µm D=0.134 & p <<0.05) while the 250 µm and 500 µm distributions were not significantly distinct (250 µm-500 µm D=0.079 & p=0.280)

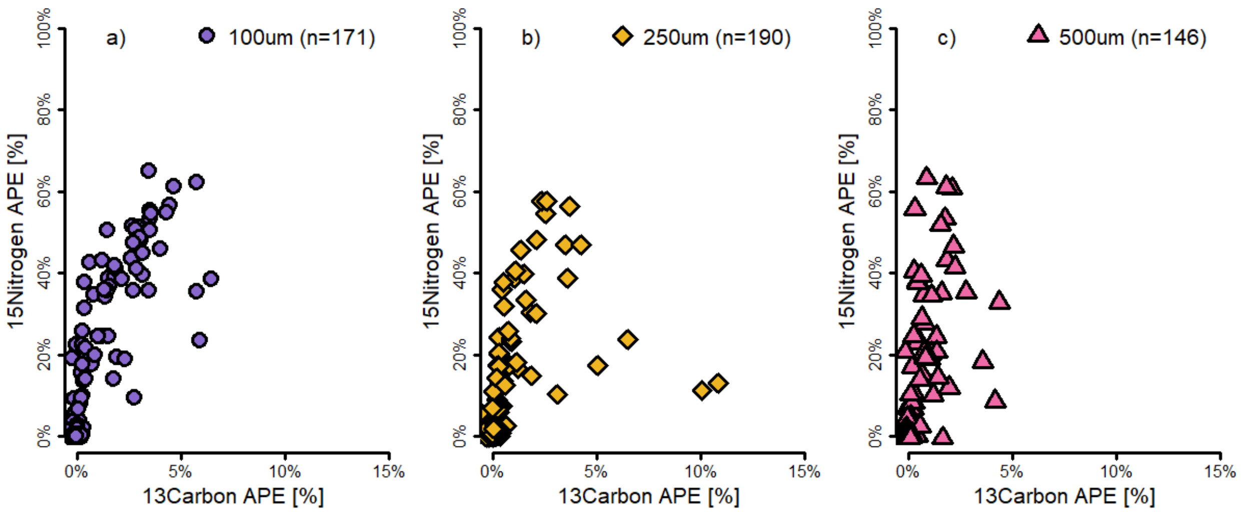


**Figure S3:** Biomass incorporation of carbon has a positive relationship with nitrogen incorporation in hydrogel microspheres inoculated with the wetland microbial community. The relationship between atom percent enrichment (APE) of $^{13}C$ and $^{15}N$ is shown for a) 100µm beads (purple circles), b) 250µm beads (yellow diamonds), and c) 500µm beads (pink triangles). The wetland microbial community was simultaneously enriched with $^{15}N$ algal protein, $^{15}N$ ammonium, and $^{13}C$ algal protein at the start of the hydrogel bead incubation. Both values were calculated as atom percent enrichment ($C_{APE}$ is directly proportional to $C_{net}$) since multiple nitrogen sources could be calculated as $N_{net}$. Calculated $C_{net}$ was multiplied by 5 due to the isotope dilution of $^{13}C$ caused by the embedding resin.

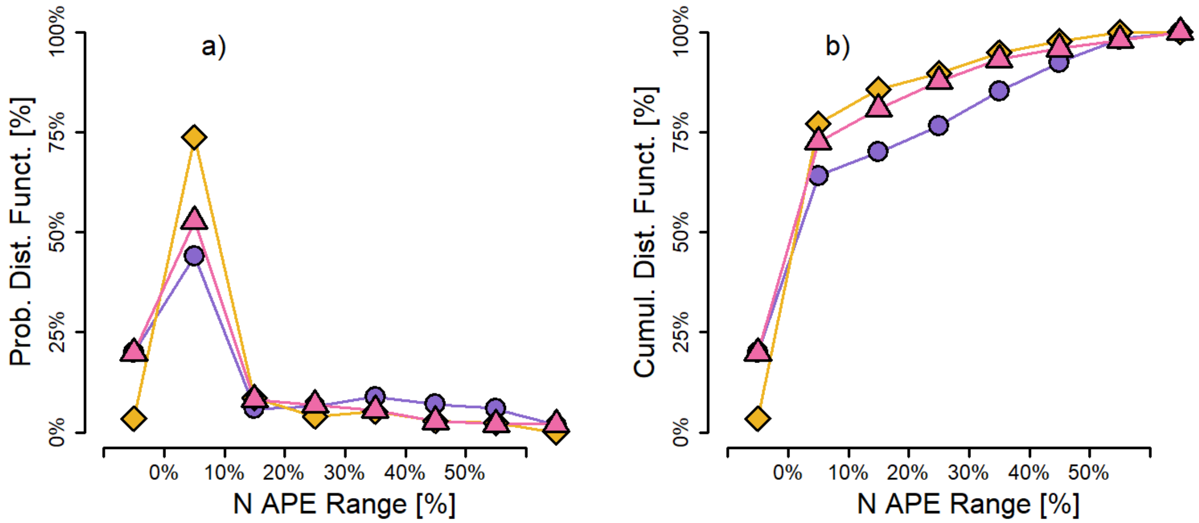


**Figure S4:** The 100 µm microspheres had a higher activity based on $^{15}N$-atom percent excess (APE). Inactive and low activity cells created a positively skewed distribution across all microsphere sizes. All incubations were given a mixture of $^{15}N$-algal protein and $^{15}N$-ammonium to detect cells via nanoSIMS, including the control cells, therefore a cutoff for active/inactive is not shown. The a) probability distribution function and b) cumulative distribution function for $^{15}N$-APE of the wetland microbial community analysis is shown with 100µm (purple circles), 250 µm (yellow diamonds) and 500 µm microspheres (pink triangles). This analysis created bins at 10% intervals to represent the percentage of analyzed cells whose $^{15}N$-APE fell within a bin's range.

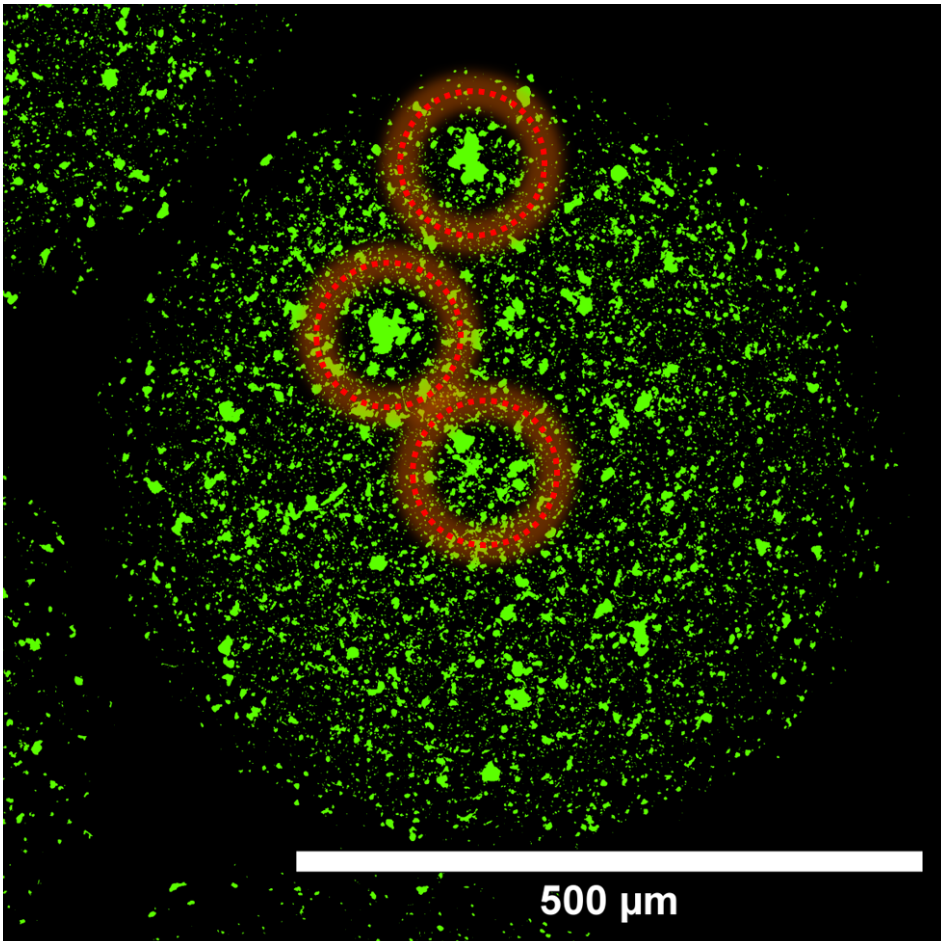


**Figure S5:** SYBR Gold stained *Flavobacterium* sp. on a z-stack maximum projection image of a 500 μm microsphere showing several regions outlined with red dashed circles, which are likely larger microporous cavities with higher abundances of microbial cells with higher activity.

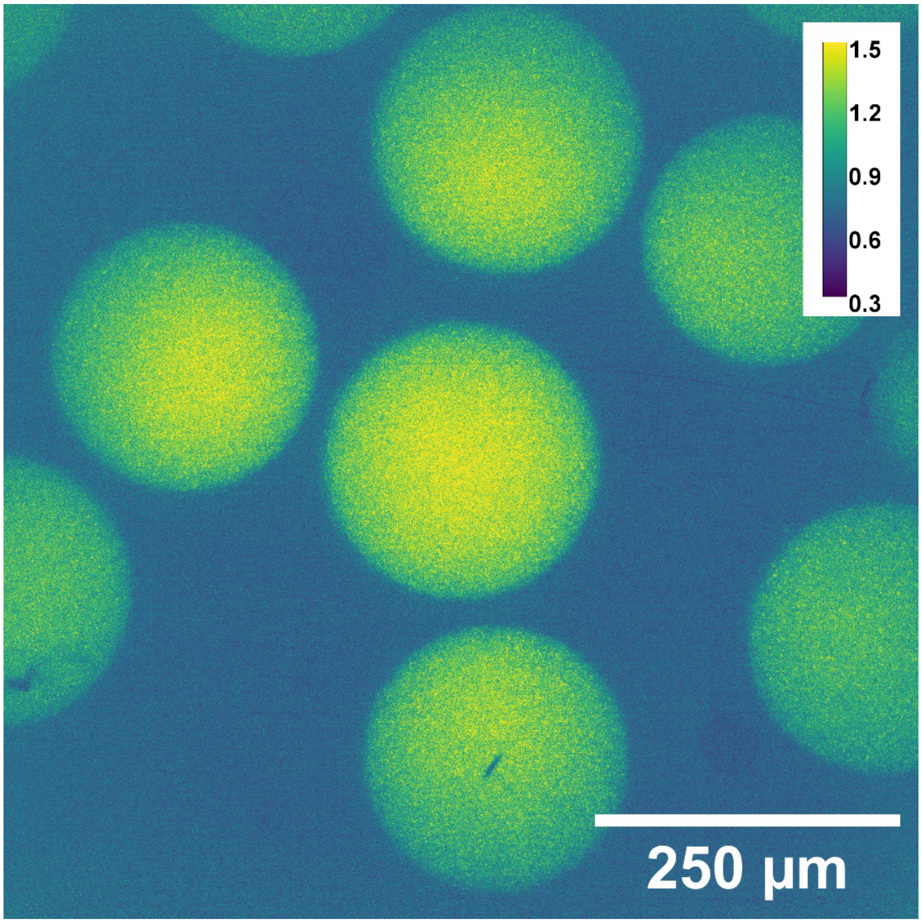


**Figure S6:** Fluorescence confocal microscopy image of a cross section through the center of several hydrogel microspheres to reveal the oxygen concentration using nanosensors. The ratio of two fluorescent signals is shown on the scale bar where the oxygen intensity reading at 405nm excitation with 640 – 660nm emission is divided by the reference signal at 405nm excitation with 435 – 485 excitation. The ratio is inversely proportional to oxygen concentration where 0.3 is at saturation (~8 mg/L of oxygen) and 1.5 is the lowest oxygen concentration of around 2.7 mg/L based on the Stern-Volmer equation.

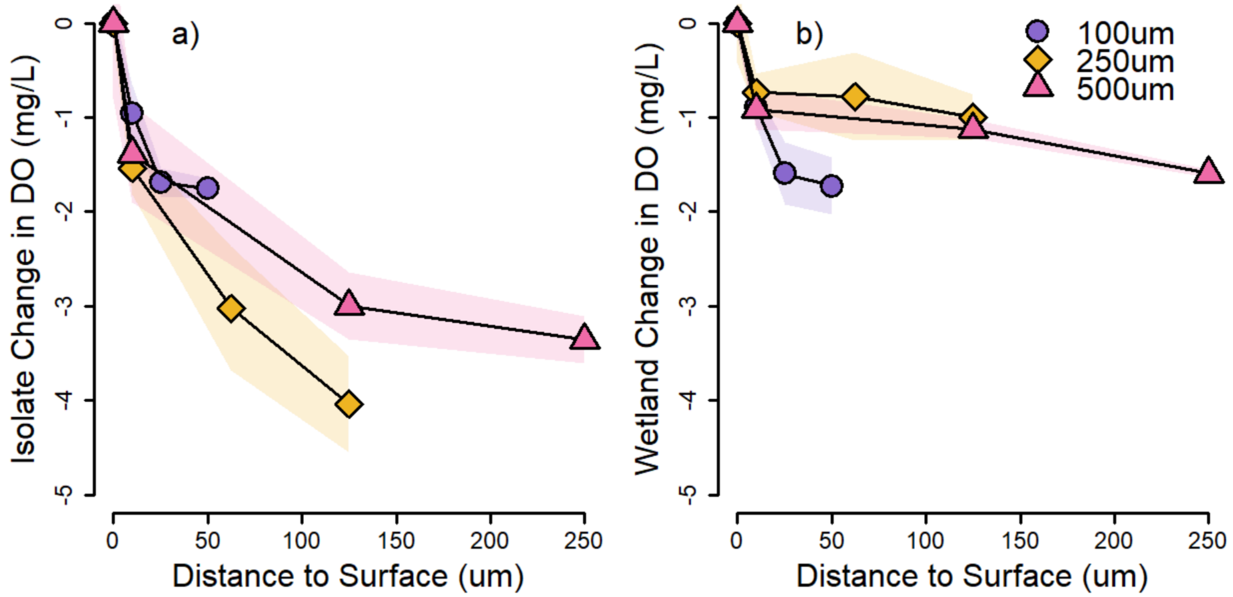


**Figure S7:** The *Flavobacterium* a) 250 µm microspheres (yellow diamonds) had a significantly lower decrease in oxygen concentration from the surface to 50 µm into the microsphere while the wetland b) 100 µm microspheres (purple circles) had a significantly lower oxygen concentration. The change in dissolved oxygen concentration is shown normalized across all microsphere diameter sizes where 0 represents the liquid/surface interface and the oxygen concentration drops moving further into the microspheres.

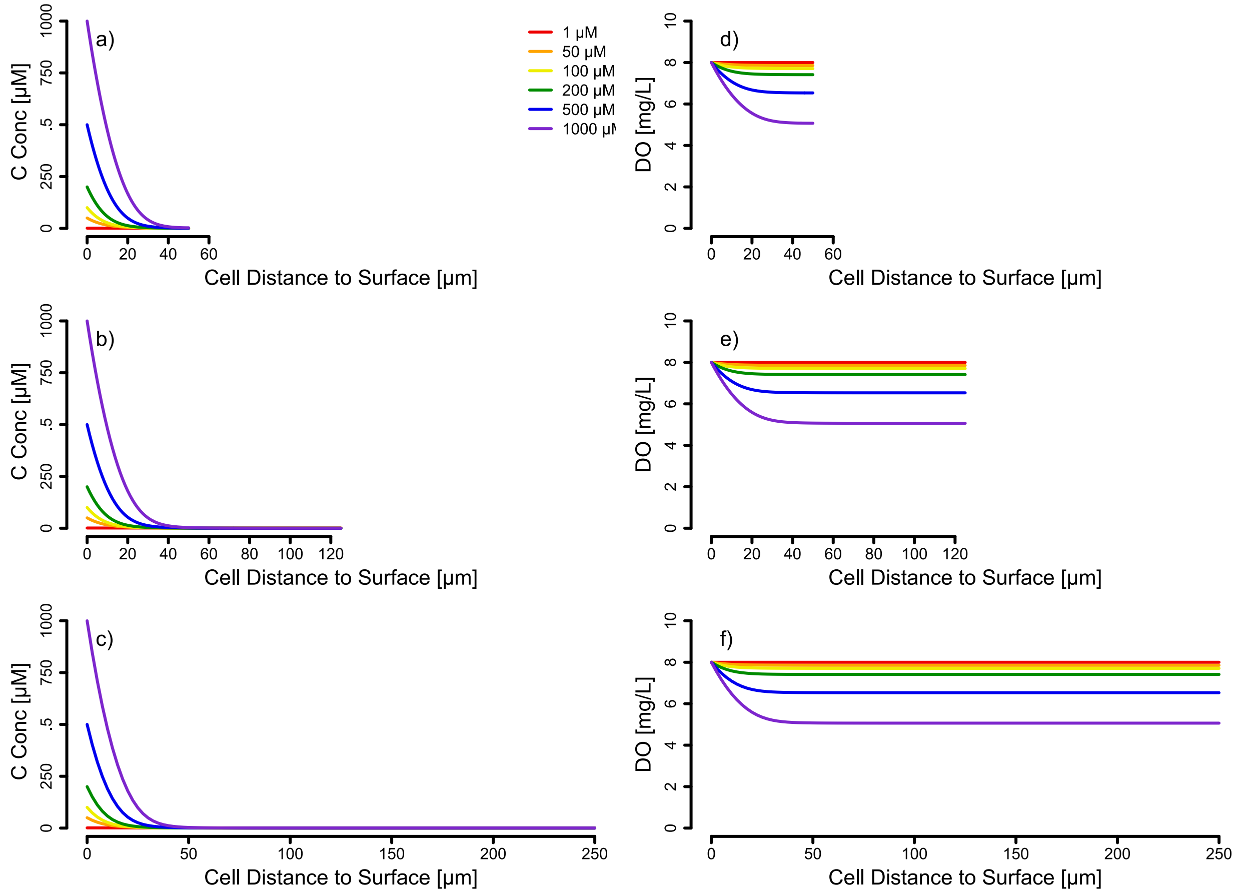


**Figure S8:** The isolate, *Flavobacterium* sp., is C-limited when modeled with varying organic C concentrations (1 μM up to 1000 μM) and never reaches oxygen limiting conditions at steady state. The model is built on a 300-day simulation of steady-state supply of C and oxygen to determine the gradients across microsphere diameters. The left-side panels model the C concentration across a) 100 μM diameter, b) 250 μM diameter, and c) 500 μM diameter microspheres. The right-side panels model the oxygen concentration within a microsphere when the population is fed each carbon concentration across a) 100 μM diameter , b) 250 μM diameter , and c) 500 μM diameter microspheres.

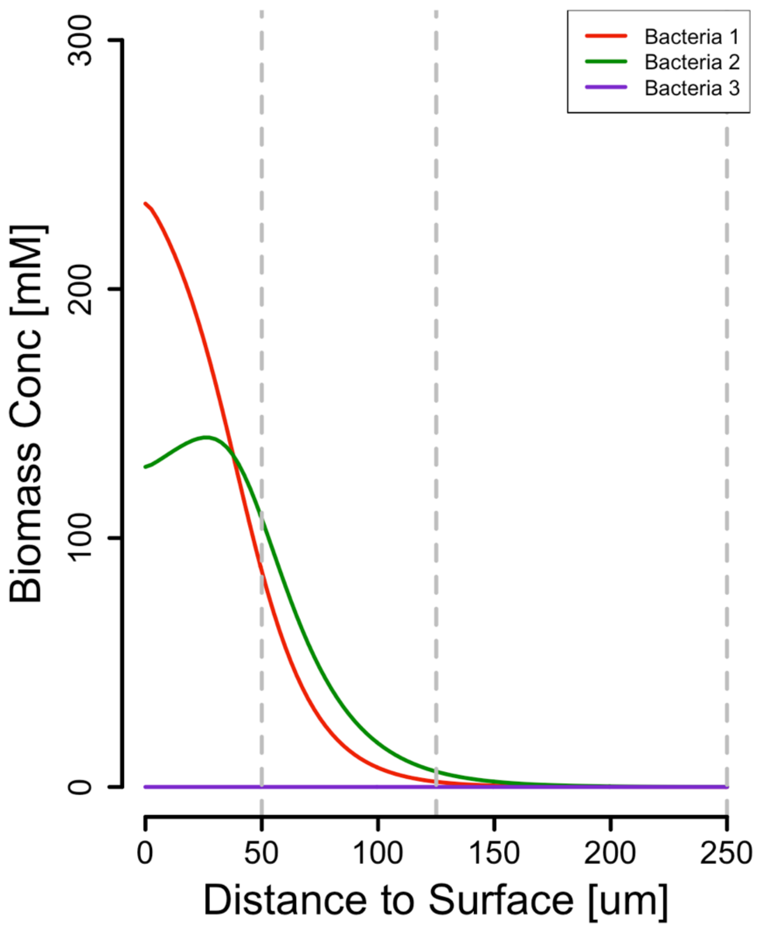


**Figure S9:** One-dimensional modeling of 3-member microbial community. Dotted lines represent the location of the center of 100 μm, 250 μm, and 500 μm diameter microspheres. The model was built on a 300-day simulation of steady-state supply of carbon and oxygen to determine the gradients of biomass across microsphere diameters.

Additional references


1. Winkler, M.K.H., et al., *Modelling simultaneous anaerobic methane and ammonium removal in a granular sludge reactor.* Water Research, 2015. **73**: p. 323-331.
2. Stewart Philip, S., *Diffusion in Biofilms.* Journal of Bacteriology, 2003. **185**(5): p. 1485-1491.
3. Picioreanu, C., M.C.M. van Loosdrecht, and J.J. Heijnen, *A new combined differential-discrete cellular automaton approach for biofilm modeling: Application for growth in gel beads.* Biotechnology and Bioengineering, 1998. **57**(6): p. 718-731.
4. Henze, M., et al., *Activated Sludge Models ASM1, ASM2, ASM2d and ASM3*. 2006, IWA Publishing.
5. Mozumder Ms Fau - Picioreanu, C., et al., *Effect of heterotrophic growth on autotrophic nitrogen removal in a granular sludge reactor.* (0959-3330 (Print)).
6. Wanner, O.E., H.J.; Morgenroth, E; Noguera, D.; Picioreanu, C.; Rittmann, B.E.; Van Loosdrecht, M.C.M., *Mathematical Modeling of Biofilms*. 2006, IWA Publishing.
7. Sabba, F., et al., *Hydroxylamine Diffusion Can Enhance N2O Emissions in Nitrifying Biofilms: A Modeling Study.* Environmental Science & Technology, 2015. **49**(3): p. 1486-1494.
